# Biomechanics of the lower limb in patients with mild knee osteoarthritis during the sit-to-stand task

**DOI:** 10.1186/s12891-024-07388-z

**Published:** 2024-04-06

**Authors:** Jing Pan, Wei Fu, Jinmiao Lv, Huiyi Tang, Zhiguan Huang, Yu Zou, Xiaohui Zhang, Bagen Liao

**Affiliations:** 1 Department of Sports Medicine, Guangzhou Sport University, Guangzhou, 510000 China; 2 School of Sports and Health, Guangzhou Sport University, Guangzhou, 510000 China

**Keywords:** Knee osteoarthritis (KOA), Sit-to-stand, STS, Elderly, biomechanics, Electromyography (EMG)

## Abstract

**Background:**

Knee osteoarthritis (KOA) is a prevalent and debilitating condition that markedly affects the sit-to-stand (STS) activity of patients, a prerequisite for daily activities. Biomechanical recognition of movements in patients with mild KOA is currently attracting attention. However, limited studies have been conducted solely on the observed differences in sagittal plane movement and muscle activation.

**Aim:**

This study aimed to identify three-dimensional biomechanical and muscle activation characteristics of the STS activity in patients with mild KOA.

**Methods:**

A cross-sectional study was conducted to observe the differences between patients with mild KOA and a control group (CG). It was conducted to observe the differences in muscle activation, including root mean square (RMS%) and integrated electromyography (items), kinematic parameters like range of motion (ROM) and maximum angular velocity, as well as dynamic parameters such as joint moment and vertical ground reaction force (vGRF).

**Results:**

Patients with mild KOA had a higher body mass index and longer task duration. In the sagittal plane, patients with KOA showed an increased ROM of the pelvic region, reduced ROM of the hip–knee–ankle joint, and diminished maximum angular velocity of the knee–ankle joint. Furthermore, patients with KOA displayed increased knee–ankle joint ROM in the coronal plane and decreased ankle joint ROM in the horizontal plane. Integrated vGRF was higher in both lower limbs, whereas the vGRF of the affected side was lower. Furthermore, patients showed a decreased peak adduction moment (PADM) and increased peak external rotation moment in the knee joint and smaller PADM and peak internal rotation moment in the ankle joint. The affected side exhibited decreased RMS% and iEMG values of the gluteus medius, vastus medialis, and vastus lateralis muscles, as well as a decreased RMS% of the rectus femoris muscle. Conversely, RMS% and iEMG values of the biceps femoris, lateral gastrocnemius, and medial gastrocnemius muscles were higher.

**Conclusion:**

The unbalanced activation characteristics of the anterior and posterior muscle groups, combined with changes in joint moment in the three-dimensional plane of the affected joint, may pose a potential risk of injury to the irritated articular cartilage.

## Introduction

Knee osteoarthritis (KOA) is a prevalent and debilitating condition characterized by pain, stiffness, weakened quadriceps, instability, and impaired functionality [1, 2]. It seriously affects patients’ quality of life, with approximately 650 million people worldwide affected in 2020 [3].

Completion of the sit-to-stand (STS) activity is closely related to the quality of life [4, 5]. In daily life, walking is a basic activity, but STS and stand-to-sit tasks are the prerequisite for and termination of gait, respectively [6]. STS movements are also prerequisites for other activities of daily living and are essential for independent living among the elderly [5]. On an average, individuals perform approximately 60 STS motions each day [7]. However, it is a complicated process that requires coordinated contraction of the lower extremity and trunk muscles and high mechanical requirements [8]. With increasing age, STS becomes more demanding for functional daily tasks [9, 10], and older people use up to 95% of their knee extensor strength when rising from a low-height chair [11].

The STS postural transition has been assessed for multiple purposes [5], such as the evaluation of fall risk, postural control, and lower extremity strength [12–15]. One of the five physical function tests recommended for people with KOA by the Osteoarthritis Research Society International (OARSI) is the 30-s Chair Stand Test (30sCST) [16]. In this test, the participant is asked to correctly perform as many stand-to-sit repetitions as possible within 30 s. Compared with a single STS task, it is easier to fully capture the impaired postural balance and biomechanical alterations [17].

Currently, extensive research has been conducted on the biomechanical characteristics of the STS task in individuals with KOA [18]. For instance, Turcot discovered a significant increase in the time required to perform the STS task in patients with KOA [19]. Furthermore, patients with KOA exhibited a increased trunk flexion angle and forward center of mass displacement during chair rising [18, 20], with men relying more on knee power and hip flexion, whereas women relied more on hip abductor strength and knee flexion angle [21]. However, these studies highlighted sagittal changes, whereas coronal and horizontal biomechanical changes are rarely studied. This may affect the identification and risk assessment of movements in patients with mild KOA.

Thus, this study aimed to analyze the three planes of lower limb kinematics and kinetics and the associated surface electromyography (sEMG) parameters of patients with mild KOA during the STS task. We expect to find valuable biomechanical clues for the mild of KOA. We hypothesize that patients with KOA exhibit a reduced range of motion in the sagittal plane but enhanced mobility in the coronal and horizontal planes. Furthermore, we anticipate an increase in muscle activation and joint moment, especially at the knee joint.

## Methods

The G*Power software (version 3.1.9.7, Franz Faul, University of Kiel) was used to determine a sample size of 36 participants. This calculation was based on an effect size of 0.8, an alpha probability of 0.05, a beta probability of 0.7, and a ratio of 2 for the KOA group to the control group (CG).

From October 2022 to April 2023, participants were recruited in Guangzhou through advertising and posters. Two distinct participant samples were recruited for this study: a KOA group and a CG. Prior to participation, all participants provided informed consent and were provided detailed information regarding the precautions of the experiment. This study was conducted as a case–control investigation and was approved by the Human Subject Committee of Guangzhou Sport University (2022LCLL-32).

The KOA group included individuals aged between 55 and 70 years, diagnosed with unilateral KOA with a Kellgren–Lawrence grade of I or II based on X-ray [22], having a visual analog scale score of ≥ 2 [23], body mass index (BMI) of < 28, and with the dominant side affected by KOA to avoid laterality interaction. A rheumatologist diagnosed KOA in accordance with the European Alliance of Associations for Rheumatology recommendations [24]. The CG, on the other hand, comprised healthy elderly individuals who were age and sex-matched and confirmed to have no pathological symptoms of KOA [24].

The exclusion criteria were as follows: knee surgery within the last 6 months, corticosteroid injection within the last 3 months, hip or ankle joint injuries, knee joint pain caused by other factors, certain primary or secondary muscle-related diseases (certain myopathies, Parkinson’s disease, muscle spasms, etc.), and mental disorders (depression, obsessive–compulsive disorder, schizophrenia/psychosis, etc.).

The 30sCST is a functional assessment that is recommended by OARSI [16] for patients with KOA. It involves using a 43-cm (17-inch) chair without armrests or back support. During the task, participants are instructed to cross their arms over their chest and perform as many STS transitions as possible within the given time frame. The initiation of the task is marked by a vertical ground reaction force of ≥ 10 N. T1 refers to the time of maximum hip flexion angle during the standing phase, whereas the end of the task is determined by the first transition of hip flexion angular velocity from negative to positive after reaching the minimum knee flexion angle [25].

**Fig. 1:**
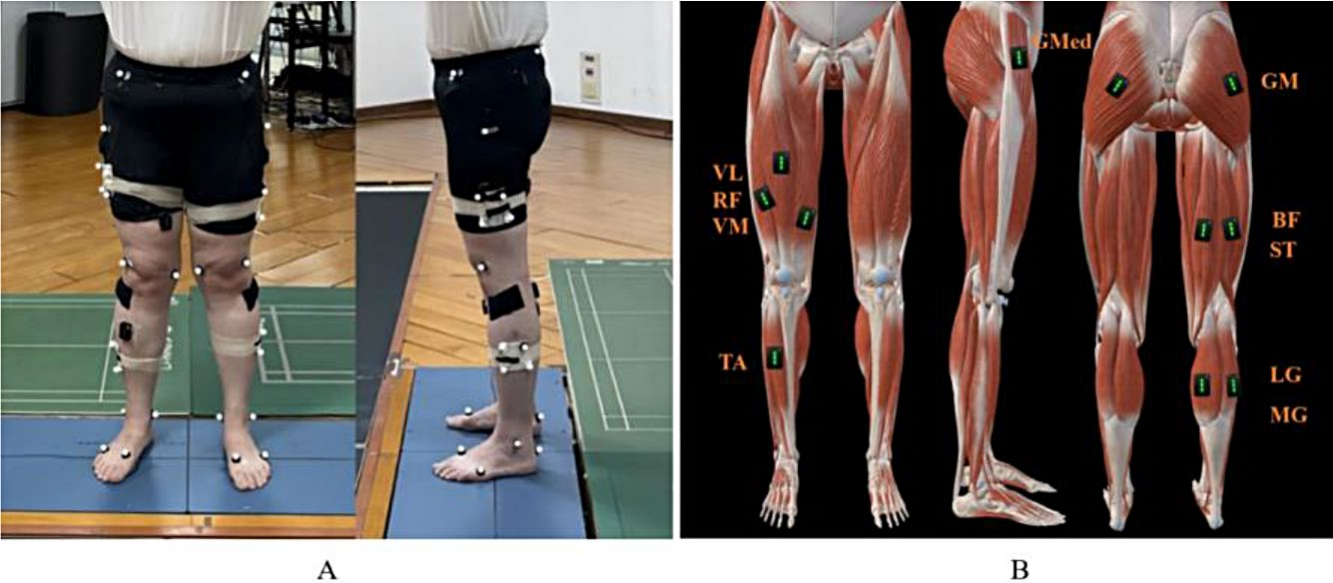
(**A**). Positions of Retroreflective Markers. IAS, Anterior superior iliac spine. IPS, Posterior superior iliac spine. TH1-4 Cluster, Cluster of four markers placed on the lateral surface of the thigh. FLE, Lateral epicondyle. FME, Medial epicondyle. SK1-4 Cluster, Cluster of four markers placed on the lateral surface of the shank. FAL, Lateral prominence of the lateral malleolus. TAM, Medial prominence of the medial malleolus. FCC, Aspect of the Achilles tendon insertion on the calcaneus. FM1, Dorsal margin of the first metatarsal head. FM5, Dorsal margin of the fifth metatarsal head. (**B**). Placement of Sensors GMed, Gluteus medius. GM, Gluteus maximus. BF, Biceps femoris. ST, Semimembranosus. LG, Lateral gastrocnemius. MG, Medial gastrocnemius. VL, Vastus lateralis. RF, Rectus femoris. VM, Vastus medialis. TA, Tibialis anterior

In this study, kinematic data were captured using a Vicon system (Nexus Vicon, Oxford, UK, containing 10 infrared induction cameras) at a frequency of 100 Hz. The Vicon system relies on retroreflective markers to recognize body movement trajectories. The placement of these markers is illustrated in Fig. 1 (A). Two force platforms (AMTI OR6-7, Watertown, MA, USA, 60 × 40 cm) were embedded in the floor to capture kinetic parameters during the task at a frequency of 1000 Hz.

Muscle contraction electrical activity and sequence data were recorded using a 16-channel wireless sEMG system (Trigno Wireless EMG System, Delsys Inc., Natick, MA, United States) at a frequency of 2000 Hz. The muscles examined are illustrated in Fig. 1 (B). Skin preparation and sensor placement were conducted in accordance with the Surface ElectroMyoGraphy for the Non-Invasive Assessment of Muscles guidelines [26].

Muscle normalization was conducted using the maximum voluntary isometric contraction test, the established gold standard procedure [27]. During each muscle normalization test, participants were guided, through visual and verbal stimuli, to gradually increase muscle strength, reach maximum effort, hold for 3–5 s, and then quickly relax [17]. The normalization test for each muscle was repeated three times, with a 60-s rest period between each test.

For patients with KOA, data were collected from the affected side. For the CG, data were obtained from the dominant leg, identified by asking participants three standard questions: (1) “Which leg would you use to kick a ball?“, (2) “Which leg would you step on a worm with?“, and (3) “Which leg would you use to draw a diamond on the ground?” [28].

Participants underwent three rounds of testing with a 60-s rest between each test. The participants conducted a practice session to minimize any initial bias and ensure proficiency in the movements before the formal testing. Data collection occurred during the stable phase of participant movements lasting 5–25 s. The Vicon system captured the data, which was then processed in Visual 3D (V6, C-motion Inc., Germantown, MD, United States). All data from the experiment were normalized to 101 data points, representing each percentage of the mission cycles from 0 to 100%.

SPSS 26.0 was used for data analysis. The Shapiro–Wilk test was used to verify data normality. Data that did not follow a normal distribution, including integrated vertical ground reaction force (vGRF), range of motion (ROM) of the pelvis in the sagittal plane, joint moments, and sEMG data, were transformed using a logarithmic transformation to approximate a normal distribution. Unpaired Student’s t-test was used to evaluate between-group differences in the demographic characteristics and result parameters. Statistical significance was set at *p* < 0.05 for all comparisons.

## Results

### Demographic data

A total of 24 patients (16 women) with mild KOA and 12 healthy individuals (8 women) matched for age and sex were enrolled. The two groups were not significantly different in terms of the demographic data, except for BMI [29] (Table 1)

**Table 1:** Descriptive participant demographics ($\bar{x}$± s)

	KOA group (n = 24)	Control group (n = 12)	P-value
Age (years)	63.63 ± 4.30	60.92 ± 3.53	0.07
Height (cm)	160.49 ± 7.99	164.08 ± 7.21	0.20
Body Mass (kg)	61.67 ± 6.85	56.82 ± 7.50	0.06
BMI (kg/m^2^)*	23.93 ± 1.94	21.01 ± 1.31	0.00
K-L grade I/grade II	5/19	\	\

BMI: body mass index

*means *p* < 0.05

### Temporal and kinematic parameters

Table 2 indicates that patients with KOA had longer task durations, however, no difference in T1 onset time was observed. In the sagittal plane, patients with KOA had a greater ROM of the pelvis, smaller ROM of the hip–knee–ankle joint, and smaller maximum angular velocity of the knee–ankle joint. Furthermore, patients with KOA had a greater knee–ankle joint ROM in the coronal plane and a smaller ankle joint ROM in the horizontal plane.

**Table 2:** Temporal and ROM Parameters between KOA and Control Group ($\bar{x}$±s)

	KOA group (n = 24)	Control group (n = 12)	P-value
Temporal Parameters			
Sit to Stand time (s)*	1.40±0.039	1.31±0.26	0.04
T1 time (%)	36.74±5.07	35.33±7.54	0.08
ROM(°) in the Sagittal plane			
Pelvis^†^*	3.09±2.18	2.61±1.42	0.04
Hip*	73.26±11.75	76.92±9.83	0.01
Knee*	83.09±7.67	86.78±9.06	0.00
Ankle*	20.62±5.55	22.33±3.94	0.00
ROM(°) in the Coronal plane			
Pelvis	26.83±7.24	25.31±7.50	0.07
Hip	6.42±3.04	6.63±2.43	0.50
Knee*	8.17±3.06	7.37±3.80	0.04
Ankle*	5.29±2.66	4.29±1.93	0.00
ROM(°) in the Horizontal plane			
Pelvis	3.79±2.27	3.43±1.45	0.13
Hip	12.46±3.44	11.49±5.39	0.09
Knee	8.42±3.42	8.78±3.43	0.36
Ankle*	5.13±1.50	5.54±1.54	0.02
Peak Angular Velocity (deg/s) in the Sagittal plane			
Hip	56.47±13.98	55.63±20.06	0.69
Knee*	176.31±38.33	191.58±37.99	0.00
Ankle*	23.15±6.63	25.88±8.95	0.01

T1 time (%): The percentage of T1 occurrence time during the STS task

*means *p* < 0.05

† means that the original data has already been logarithmically transformed

### Kinetic parameters

**Fig. 2:**
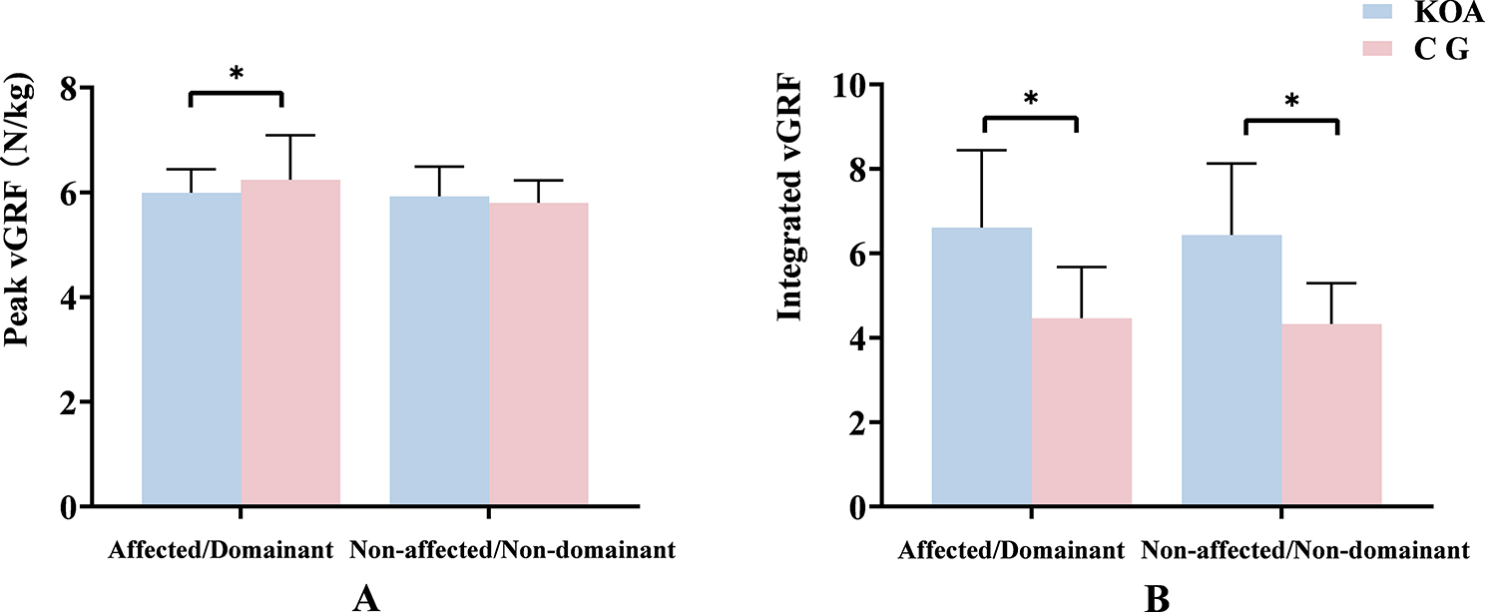
Comparison of vGRF parameters between the KOA group and the CG (± MDE). *means *p* < 0.05

**Fig. 3:**
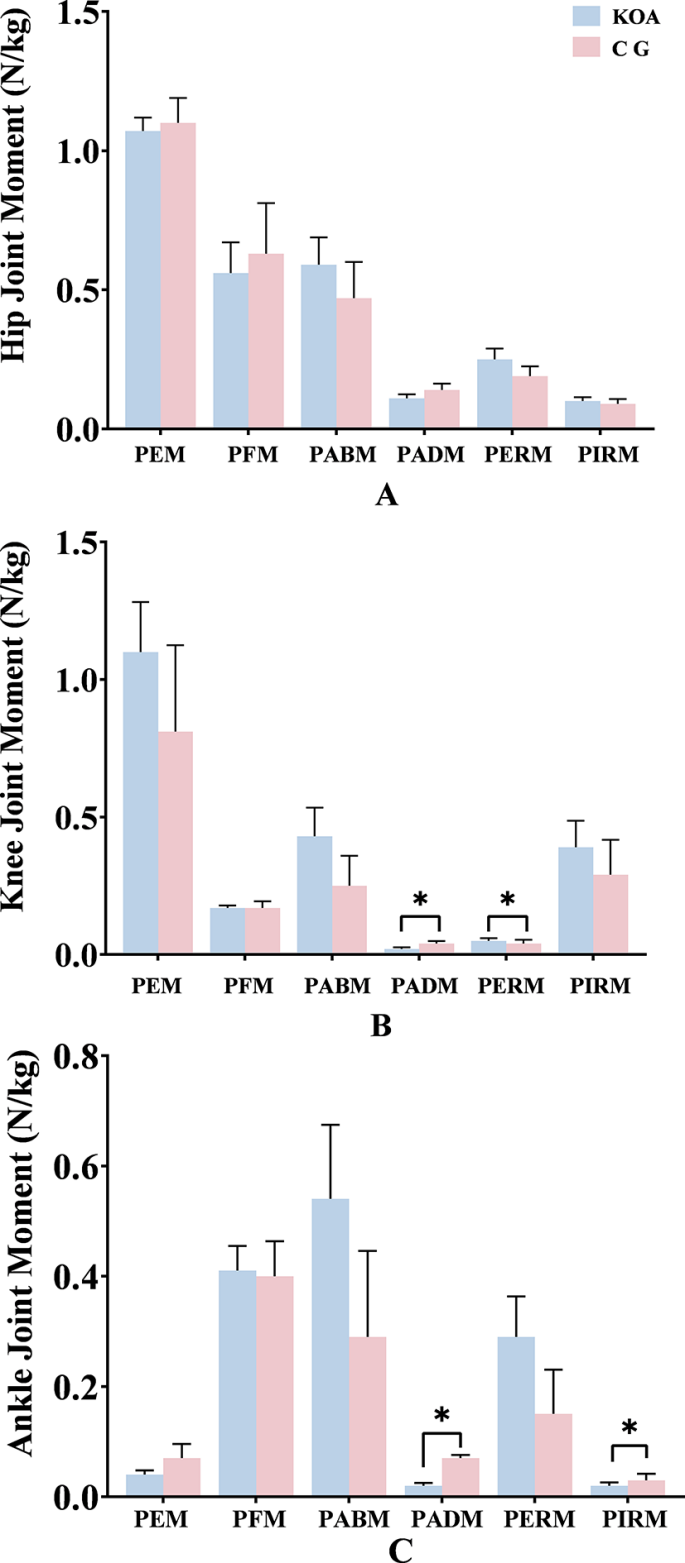
Comparison of Joint Moments Between the KOA Group and the CG ( ± MDE). All data were subjected to logarithmic transformation before statistical analysis. *means *p* < 0.05. (**A**), Hip Joint Moment; (**B**), Knee Joint Moment; (**C**), Ankle Joint Moment. PEM: peak extension moment; PFM: peak flexion moment; PABM: peak abduction moment; PADM: peak adduction moment; PERM: peak external rotation moment; PIRM: peak internal rotation moment

During the STS task, patients with KOA had a higher integrated vGRF in both lower limbs compared with the CG, and the vGRF of the affected side was lower (Fig. 2). Additionally, during the STS task, patients with KOA had a smaller peak adduction (PADM) and larger peak external rotation moment (PERM) in the knee joint, whereas the PADM and peak internal rotation moment (PIRM) of the ankle joint were smaller. However, there were no significant changes in the hip joint moment of the two groups (Fig. 3).

### sEMG data

**Fig. 4:**
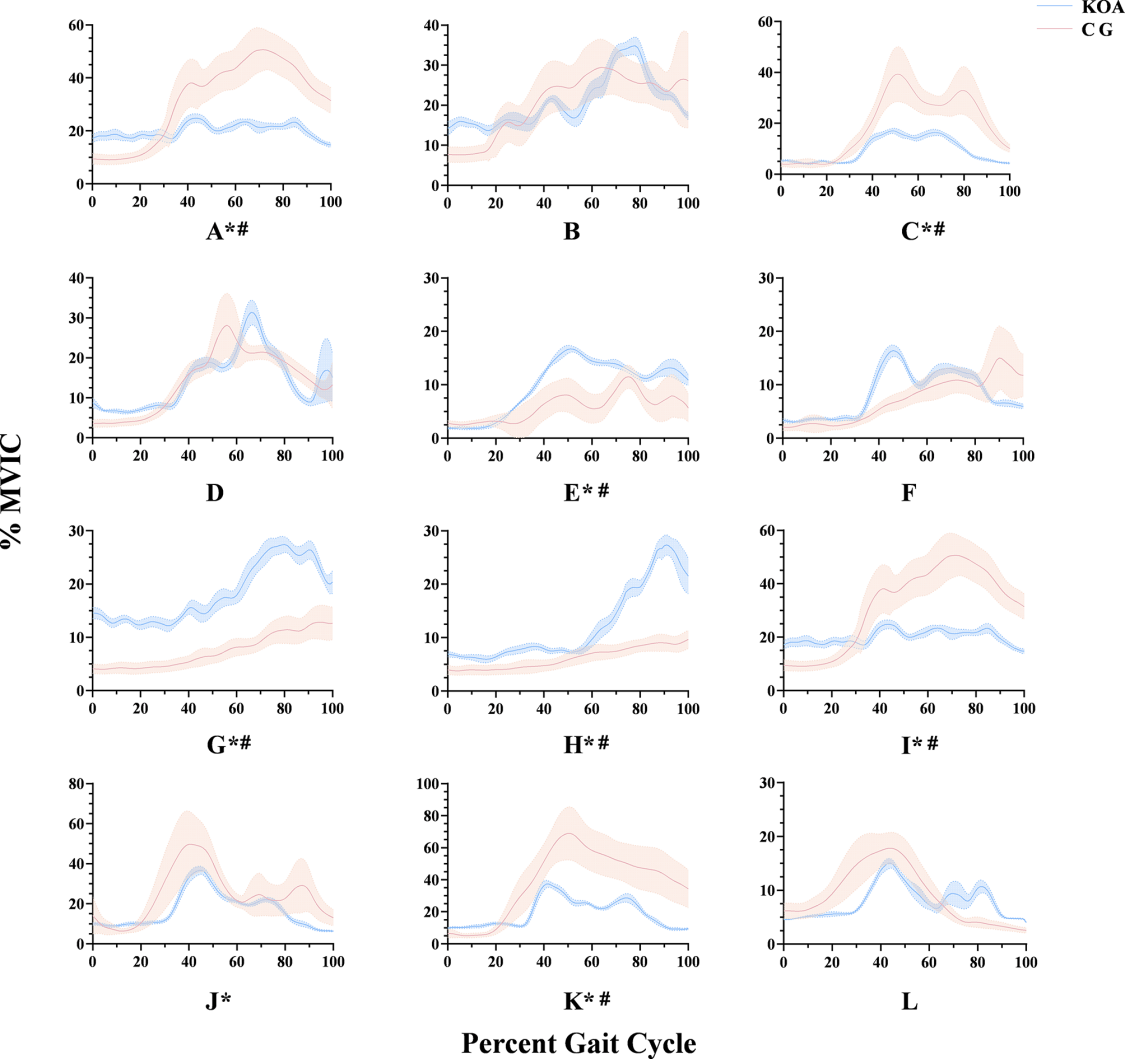
Comparison of muscle activation level between the KOA group and the CG (± MDE). All data were subjected to logarithmic transformation before statistical analysis. * mean RMS% between the two groups is p < 0.05. # mean integrated EMG (iEMG) between the two groups is p < 0.05. (**A**), Gluteus medius (GMed) of affected side; (**B**), Gluteus medius (GMed) of non-affected side; (**C**), Gluteus maximus (GM) of affected side; (**D**), Gluteus maximus (GM) of non-affected side; (**E**), Biceps femoris (BF); (**F**), Semimembranosus (ST); (**G**), Lateral gastrocnemius (LG); (**H**), Medial gastrocnemius (MG); (**I**), Vastus lateralis (VL); (**J**), Rectus femoris (RF); (**K**), Vastus medialis (VM); (**L**), Tibialis anterior (TA)

Compared with the CG, the affected side of patients with KOA had a smaller root mean square percentage (RMS%) and integrated EMG (iEMG) values of the gluteus maximus (GM), gluteus medius (GMed), vastus lateralis, and vastus medialis muscles and a smaller RMS% of the rectus femoris (RF) muscle (Fig. 4). However, the RMS% and iEMG values of the biceps femoris (BF), lateral gastrocnemius (LG), and medial gastrocnemius (MG) muscles were all higher.

## Discussion

The STS task, a pivotal activity for upright mobility [8], significantly influences functional mobility, quality of life, and independence in patients with KOA [7, 9, 30]. This cross-sectional investigation aimed to elucidate the biomechanical alterations across three planes and assess muscle activation disparities during the STS task in KOA patients. Findings indicated that KOA patients required extended durations for task completion, demonstrating diminished biomechanical adaptations in the sagittal plane and augmented modifications in the coronal plane, as evidenced by kinematic parameters. Additionally, variations in muscle activation levels and joint moments were evident between the cohorts.

Consistent with preceding research [18, 19], the extended task duration corroborates with limitations in physical functionality [21], serving as an indicator of functional mobility deficits [31] and an elevated fall risk [13]. Turcot et al.‘s study underscores a significant association between pain intensity and the average STS task completion time [19]. This extended duration and delayed T1 onset potentially mitigate pain, necessitating prolonged muscle activation for joint stiffness maintenance.

Echoing earlier findings [18, 32], the results of this study further reveal that KOA patients exhibit reduced ROM across all lower limb joints, barring the pelvis, and diminished maximum angular velocities at the knee and ankle joints. As highlighted by Patsika, diminished knee and ankle angular velocities in KOA patients are intricately linked to compromised strength, particularly within the knee extensor muscles [33]. Knee extensors have been identified as playing a paramount role, surpassing other major lower limb muscles in STS and lower extremity muscle activities [34, 35], with quadriceps weakness being a common manifestation in KOA patients [36]. Moreover, the observed sagittal pelvic ROM reduction may relate to decreased RF muscle strength. Prior research indicates an inverse relationship between knee extensor strength and trunk inclination [37], necessitating greater pelvic flexion angles to compensate for inadequate knee extensor strength [38], thereby minimizing knee exertion during the transition from sitting to standing [20].

In contrast, KOA patients were found to have heightened BF muscle activation compared to the CG, aligning with previous studies [33, 39]. This could lead to significantly increased co-contraction of knee muscles in the affected limb versus the CG [4], suggesting muscle co-contraction as a bilateral stabilization strategy. While elevated coactivation levels enhance joint stability and mitigate pain from extreme joint positions [40], they also amplify knee joint compressive loads [41, 42], potentially accelerating KOA’s structural progression [43]. The result of this study also concurs with the observation that except for the PADM, patients with KOA have greater knee joint moment than the CG. Additionally, the decreased ROM of the lower limb joints in the sagittal plane may be related to the higher co-contraction of the knee muscles [39].

Moreover, KOA patients displayed reduced peak vGRF on the affected side, with an increasing tendency on the contralateral side, relative to the CG. This aligns with studies showing patients with severe unilateral KOA bearing greater loads on the unaffected limb [19, 44], possibly due to pain avoidance behaviors [45], leading to greater lateral trunk flexion [19] and reduced PADM of the knee and ankle joints on the affected side. Notably, in this study, patients showed increased bilateral integrated vGRF, possibly due to the prolonged task duration, with significantly higher knee flexion and adduction moments on the unaffected side [44], indicating KOA’s progressive impact on the contralateral limb. It is well known that contraction of the hip extensor muscle is essential for maintaining trunk stability and controlling the speed of movement [46]. The results of this study show that patients with KOA have lower activation levels in the GMed and GM muscles of the affected lower limb, which could be attributed to the fact that the CG had a greater movement speed and more weight-bearing on their dominant side, resulting in a stronger activation of the GM and GMed muscles [47].

In the coronal plane, KOA patients demonstrated an increased ROM at the knee and ankle joints but reduced PADM during movement compared to controls. An increased ROM suggests compromised balance, a consequence of asymmetric loading [47]. Enhanced BF, LG, and MG muscle activation could stiffen the knee joint, potentially escalating knee muscle co-contraction and joint damage [41, 42]. On the axial plane, reduced ROM, PIRM of the ankle, and increased PERM of the knee were observed. Previous studies have noted the importance of tibialis anterior (TA) muscle contraction for foot stabilization and forward trunk movement [48, 49], with gastrocnemius and TA muscle co-contraction being crucial for balance and ankle stability [50, 51]. Abnormal ROM and joint moment in the ankle joint may be associated with impaired LG and MG muscle activation.

This study posits that milder damage in participants, resulting in less pronounced biomechanical parameter differences on the horizontal plane, could be due to smaller movements and all participants being first- or second-degree sufferers. Experimental outcomes suggest that patients with mild KOA may adopt asymmetric loading patterns on the lower limbs to circumvent pain, leading to altered joint moments, ROM, and muscle activation levels, thereby exacerbating biomechanical KOA progression risks, and affecting contralateral limb disease.

Compared to previous studies, this research systematically explores biomechanical alterations in mild KOA patients across three planes, incorporating muscle activation levels. Nonetheless, the absence of sex differentiation due to a limited sample size and ungraded KOA severity limits detailed insights into KOA’s early alterations. These observations underscore the critical nature of biomechanical changes and muscle activation discrepancies during the STS task in KOA patients, shedding light on their functional limitations and adopted strategies. Such insights are invaluable for crafting effective interventions to enhance KOA patients’ functional mobility and life quality.

## Data Availability

The datasets generated and/or analyzed during the current study are not publicly available due to privacy issues. However, data will be available upon formal request to the corresponding authors.

## Abbreviations

KOA: Knee osteoarthritis
CG: Control Group
ROM: range of motion
sEMG: Surface Electromyography
RMS: Root Mean Square
PEM: peak extension moment
PFM: peak flexion moment
PABM: peak abduction moment
PADM: peak adduction moment
PERM: peak external rotation moment
PIRM: peak internal rotation moment
GMed: Gluteus medius
GM: Gluteus maximus
BF: Biceps femoris
ST: Semimembranosus
LG: Lateral gastrocnemius
MG: Medial gastrocnemius
VL: Vastus Lateralis
RF: Rectus femoris
VM: Vastus Medialis
TA: Tibialis anterior
